# Microbial Screening Reveals Oral Site-Specific Locations of the Periodontal Pathogen *Selenomonas noxia*

**DOI:** 10.3390/cimb43010029

**Published:** 2021-06-12

**Authors:** Jaydene McDaniel, Steven McDaniel, Beanca Jhanine Samiano, Matthew Marrujo, Karl Kingsley, Katherine M. Howard

**Affiliations:** 1 Department of Advanced Education in Pediatric Dentistry, School of Dental Medicine, University of Nevada-Las Vegas, 1700 W. Charleston, Las Vegas, NV 89106, USA; mcdanj1@unlv.nevada.edu (J.M.); mcdans1@unlv.nevada.edu (S.M.); 2 Department of Clinical Sciences, School of Dental Medicine, University of Nevada-Las Vegas, 1700 W. Charleston, Las Vegas, NV 89106, USA; samiab1@unlv.nevada.edu (B.J.S.); marrujo@unlv.nevada.edu (M.M.); 3 Department of Biomedical Sciences, School of Dental Medicine, University of Nevada-Las Vegas, 1001 Shadow Lane, Las Vegas, NV 89106, USA; katherine.howard@unlv.edu

**Keywords:** *Selenemonas noxia*, periodontal, oral, saliva, screening

## Abstract

Introduction: *Selenomonas noxia* (SN) is an important periodontal pathogen, associated with gingivitis and periodontitis. Many studies have found associations between SN and indicators of poor health outcomes, such as smoking, low socioeconomic status and obesity. However, less is known about the prevalence of this organism and more specifically about other oral site-specific locations that may harbor this organism. Methods: Using an existing patient repository (*n* = 47) of DNA isolated from saliva and other oral sites (*n* = 235), including the dorsum of the tongue, lower lingual incisor, upper buccal molar and gingival crevicular fluid (GCF), molecular screening for SN was performed. Screening results were analyzed for associations between demographic variables (age, sex, race/ethnicity) and clinical information (body mass index or BMI, presence of orthodontic brackets, primary/mixed/permanent dentition). Results: qPCR screening revealed a total of *n* = 62/235 sites or 26.3% harboring SN with saliva and GCF (either alone or in combination with one or more sites) most often observed (Saliva, *n* = 23/27 or 85.18%, GCF, *n* = 14/27 or 51%). Analysis of site-specific data revealed most positive results were found among saliva and GCF alone or in combination, with fewer positive results observed among the tongue (33.3%), lower lingual incisor (29.6%), and upper buccal molar (25.9%). No significant associations were found between demographic or clinical variables and presence of SN at any site. Conclusions: These results may be among the first to describe site-specific locations of *S. noxia* among various additional oral biofilm sites. These data may represent a significant advancement in our understanding of the sites and locations that harbor this organism, which may be important for our understanding of the prevalence and distribution of these organisms among patients of different ages undergoing different types of oral treatments, such as orthodontic treatment or therapy.

## 1. Introduction

*Selenomonas noxia* (SN) is an anaerobic, gram-negative, crescent shaped bacterium found in the oral cavity and gastrointestinal tract of both humans and other mammalian species [1,2]. Early studies reported that this organism aggregates with many known periodontal pathogens, including *Fusobacterium nucleatum* (FN) and *Porphyromonas gingivalis* (PG), and is most strongly associated with the microbial profile of patients with gingival and periodontal disease [3,4]. Molecular profiling of this organism revealed this gingival crevice and periodontal pocket pathogen may be a leading indicator for the microbial “switch” from normal to inflamed tissue and a potential biomarker for the onset of aggressive gingivitis and periodontitis [4,5,6].

More detailed comparative analyses of elderly and periodontal patients, as well as those with Papillon–Lefevre syndrome (a rare form of precocious, early-onset pediatric periodontitis) found strong associations with SN prevalence, as well as site-specific bacterial levels within the gingival crevice and periodontal pockets [7,8,9]. Comprehensive analysis of risk factors for development of gingivitis and periodontal disease, such as smoking, low socioeconomic status and reduced access to health services and information have revealed these factors also influence SN prevalence [10,11].

Additional factors that may influence the prevalence of SN may include the placement of orthodontic brackets [12]. This may alter the behavioral and dietary patterns for adolescents and young adults, thereby increasing the frequency of gingivitis and prevalence of periodontal organisms, such as FN, PG and SN [13]. In fact, the clustering of these microbes in both supragingival and subgingival biofilms has been observed in these patient populations [14].

More recent evidence has now suggested that SN may also be associated with other adverse health outcomes, such as obesity [15]. One study found that 98.4% of obese women could be identified based upon the presence of this one bacterial species (SN) in amounts greater than 1.05% of total salivary bacteria. As this organism is capable of fermenting “indigestible” carbohydrates and extracting additional calories from fiber-containing foods, this may suggest that routine screening of saliva for this organism may provide significant clinical information regarding oral and systemic health risks among dental patients in routine care [16,17].

However, the presence of this bacteria in samples of unstimulated saliva suggests that SN may be present not only in gingival crevices and periodontal pockets but also in other biofilms and oral sites, such as the supragingival biofilm [18,19]. Although studies from this group have attempted to identify patients with oral SN from unstimulated saliva, no studies to date have evaluated the site-specific oral locations of SN within the oral cavity [20,21]. In addition, some studies have demonstrated that specific biologic determinants, such as age and sex, may influence the presence of oral biofilms and their specific constituents, which may provide much needed contextual and correlated information [22,23].

Based upon this information, the primary goal of this study was to determine which specific oral sites other than the gingival crevice and saliva may harbor this organism, including the dorsum of the tongue, lingual surfaces of the mandibular incisor, and buccal surface of the maxillary molars using an existing patient repository. The secondary objectives of this study were to determine if any of patient demographics (age, sex, race/ethnicity) or clinical information (body mass index or BMI, permanent, primary or mixed dentition, brackets or orthodontic appliances) were associated with these SN results.

## 2. Materials and Methods

### 2.1. Human Subjects

This retrospective study of existing DNA isolated from clinical saliva samples was reviewed and approved exempt by the Office for the Protection of Research Subjects (OPRS) and Institutional Review Board (IRB) at the University of Nevada, Las Vegas (UNLV) #1717625-1 titled “Retrospective analysis of microbial prevalence from DNA isolated from saliva samples originally obtained from the University of Nevada, Las Vegas (UNLV) School of Dental Medicine (SDM) pediatric and clinical population”. The original study protocol under which the saliva samples were collected was approved under protocol OPRS#1305-4466M “The Prevalence of Oral Microbes in Saliva from the UNLV School of Dental Medicine Pediatric and Adult Clinical Population” in 2013.

### 2.2. Study Protocol

This study was a retrospective study of previously collected saliva and oral samples. In brief, in the original study sample collection protocol adult patients over 18 years of age were asked to participate and provide Informed Consent. Pediatric patients over the age of seven but under the age of 18 were asked to participate and provide Pediatric Assent. In addition, a parent or guardian for each pediatric patient was also asked to provide Informed Consent. Inclusion criteria included all patients willing to participate in the voluntary study of microbial prevalence who provided Informed Consent or Pediatric Assent. Exclusion criteria included all patients who declined to participate or declined to provide Informed Consent or Pediatric Assent. No patients were excluded due to the presence of systemic diseases or their related conditions and associated medications.

### 2.3. Sample Collection

In the original study collection protocol, saliva samples were collected in sterile tubes (up to 5 mL) and were labeled with a randomly generated, non-duplicated number to prevent any patient identifying information from being associated with any sample. In addition, paper points were used to collect gingival crevicular fluid (GCF) from the central maxillary incisor and biofilm samples from the dorsum of the tongue, lingual surface of the mandibular incisors and buccal surface of the maxillary molars. All samples were collected at the beginning of the randomly chosen clinic session, prior to any hygiene or other clinical treatment protocol. All samples were stored on ice and transferred within two hours to a biomedical laboratory for storage at −80 °C for processing.

Finally, basic demographics (age, sex, race/ethnicity) and clinical information (body mass index, permanent, primary or mixed dentition, brackets or orthodontic appliances) were collected in the original protocol for subsequent analysis. Due to the nature of the original saliva sampling study objectives, additional clinical information such as decayed-missing-filled teeth (DMFT) score, smoking status, periodontal disease and periodontal pocket depth (PPD) scores were not collected and were therefore not available for analysis in the current retrospective analysis.

### 2.4. DNA Isolation

DNA was extracted from 100 μL of each saliva sample using the phenol: chloroform extraction method using TRIzol reagent from Invitrogen (Waltham, MS, USA). DNA was extracted from paper points by adding 100 μL of sterile 1X phosphate buffered saline (PBS) to each tube and vortexing the tube to release any collected materials. TRIzol from ThermoFisher (Fair Lawn, NJ, USA) was then added to each tube (300 μL) and the extraction method was completed according to the manufacturer protocol, as previously described [24]. In brief, 200 μL of chloroform was added to each tube and then centrifuged at 12,000× *g* (relative centrifugal force, RCF) for 15 min at 4 °C. The supernatant was removed and placed into a new micro centrifuge tube with 100 μL of isopropanol to precipitate the DNA. Samples were then centrifuged to pellet the DNA, which was then washed with ethanol prior to resuspension in 100 μL of sterile, nuclease-free water.

### 2.5. DNA Screening

DNA isolates were screened using a NanoDrop spectrophotometer from ThermoFisher (Fair Lawn, NJ, USA) at absorbances of A260 nm and A280 nm to determine the quality and quantity. Absorbance readings at A260 were used to determine the quantity, adjusting for the dilution factor. A minimum of 10 ng was required for each sample to be included in the current study. The ratio of absorbance readings at A260:A280 were used to determine the sample quality, with minimum sample ratios set at the ratio of 1.7.

### 2.6. qPCR (Quantitative Polymerase Chain Reaction)

Each sample with sufficient DNA concentration (10 ng) and sufficient quality (A260:A280 > 1.7) was then identified for inclusion in this study. Screening for the presence of microbial DNA was performed using primers specific to this organism, *Selenomonas noxia* (SN), as previously described [16,20,21]. In brief, qPCR was performed using ABsolute SYBR green (no ROX) from ThermoFisher (Fair Lawn, NJ, USA). Each reaction consisted of 12.5 μL of 2X ABsolute SYBR green master mix, 1.75 μL of forward primer, 1.75 of reverse primer, 1.5 μL of sample (diluted to 1.0 ng/μL) and 7.5 μL of distilled, nuclease-free water. Each reaction was performed using enzyme activation for 15 min at 95 °C followed by 40 cycles of denaturation for 15 s at 95 °C, annealing at the primer specific temperature of each primer pair for 30 s and extension at 72 °C for 30 s.

#### Positive Control 16S rRNA Universal Primer

Forward 16S rRNA universal primer, 5′-ACG CGT CGA CAG AGT TTG ATC CTG GCT-3′

27 nt, 56% GC, Tm 76 °C

Reverse 16S rRNA universal primer, 5′-GGG ACT CAG TAT TAT-3′

21 nt, 48% GC, Tm 62 °C

Annealing temperature for primer set: 57 °C

### 2.7. Selenomonas noxia (SN) Primer

Forward primer- SN-F1, 5′-TCT GGG CTA CAC ACGT ACT ACA ATG-3′

25 nt, 48% GC, Tm: 68 °C

Reverse primer- SN-R1, 5′-GCC TGC AAT CCG AAC TGA GA-3′

20 nt, 55% GC, Tm: 68 °C

Annealing temperature for primer set: 63 °C

### 2.8. Statistical Analysis

Descriptive statistics were compiled for all demographic variables (age, sex, race/ethnicity) and comparisons were made with the overall clinic demographics using Chi square analysis from GraphPad (San Diego, CA, USA). DNA concentration and other parametric measurements were compiled and descriptive statistics provided. Analysis of these data was performed using analysis of variance (ANOVA) using Microsoft Excel (Redmond, WA, USA).

## 3. Results

A total of N = 47 patient samples were available from the existing repository for analysis and screening in the current study (Table 1). In brief, the sample demographics revealed slightly less than half of samples were derived from females (42.6%), which was not significantly different from the overall demographics of the pediatric clinic from which the samples were originally taken (*p* = 0.2300). The racial and ethnic breakdown of the study sample was mostly derived from minority (non-White) patients (83%), which was significantly higher than the overall percentage within the clinic (65.4%) (*p* = 0.0002).

**Figure 1:**
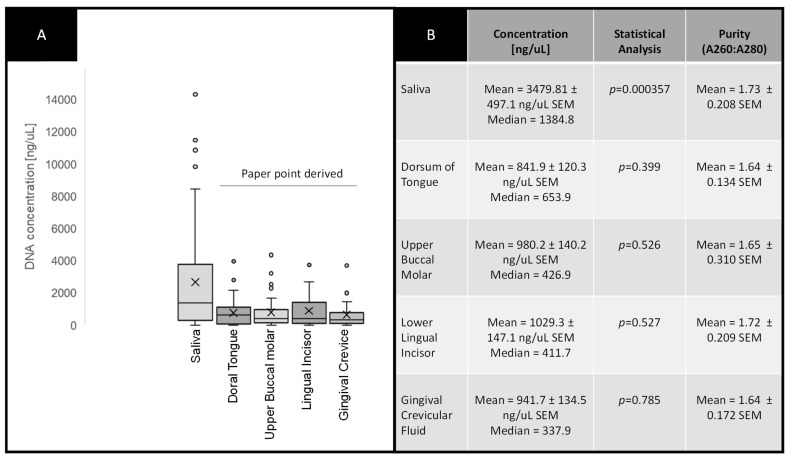
Analysis of DNA isolation. (**A**) Box-and-whisker plot of DNA concentrations from saliva and paper point sampling sites (dorsal tongue, upper buccal molar, lingual incisor, gingival crevice) demonstrates higher averages among saliva samples (*p* = 0.000357). (**B**) Average DNA concentrations from paper point sampling sites were not significantly different from one another (*p* = 0.399).

DNA isolated from each of the five sites from each patient sample was analyzed for purity and concentration (Figure 1). More specifically, data were organized to create box-and-whisker plots to graphically display the concentrations of DNA isolated from patient saliva and paper point-derived samples using the Tukey method to visualize the mean, median and 1st and 3rd interquartile ranges (Figure 1A), which are also provided in summary form (Figure 1B). These data revealed that average DNA concentrations from saliva (Ave. =3479.81 ng/μL) were significantly higher than those derived from paper points (Ave. = 948.29 ng/μL) (*p* = 0.000357). However, the DNA samples isolated from paper points derived from various sites, including the tongue (Ave. = 841.0 ng/μL), upper buccal molar (Ave. = 980.2 ng/μL), lower lingual incisor (Ave. = 1029.3 ng/μL) and gingival crevicular fluid (Ave. = 941.7 ng/μL) were not significantly different from one another or the average of all combined paper point derived samples (Ave. = 948.29 ng/μL) (*p* = 0.399).

**Figure 2:**
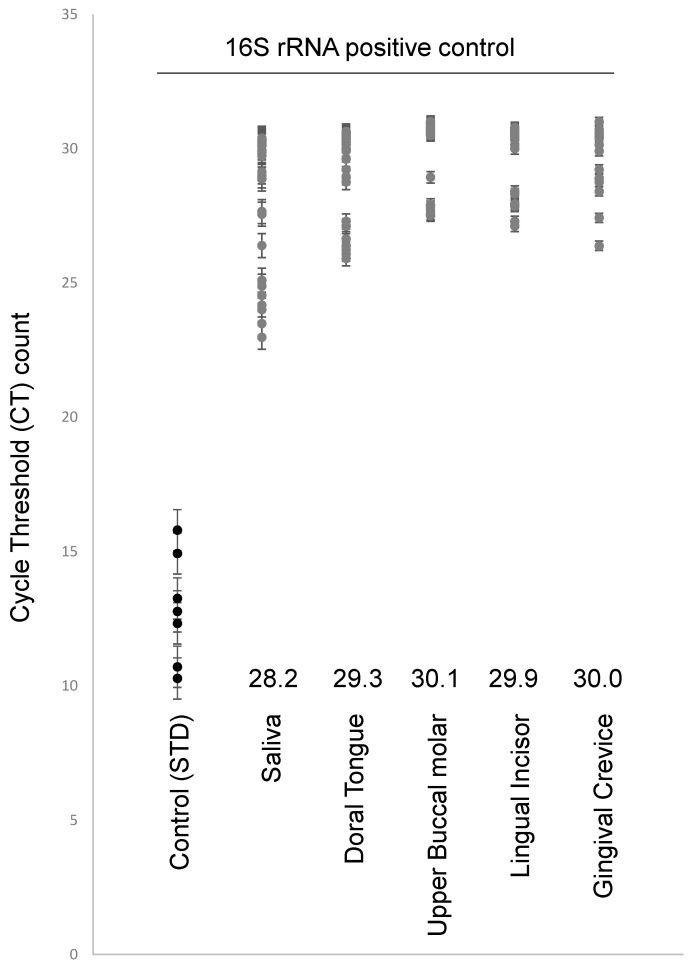
Real time qPCR screening of samples for 16S rRNA. Positive control standard 16S rRNA revealed all study samples harbored bacterial DNA with average cycle threshold (CT) counts ranging from 28.2 to 30.1, which were not significantly different from one another (*p* = 0.513) but were higher than the positive control standards (10 ng/μL, 5 ng/μL, 2 ng/μL; CT average =12.86) (*p* = 0.00018).

In order to verify the presence of bacterial DNA from each sample, real time quantitative polymerase chain reaction (RT-qPCR) was performed using 16S rRNA (Figure 2). These data demonstrated that all samples harbored DNA of sufficient quantity and quality for this analysis. The cycle threshold (CT) average count for the study samples was significantly higher (CT range: 28.2–30.1) than the average CT count for the known bacterial standards at 10 ng/μL, 5 ng/μL, and 2 ng/μL (CT average 12.86) (*p* = 0.00018), but not significantly different from one another (*p* = 0.513).

**Figure 3:**
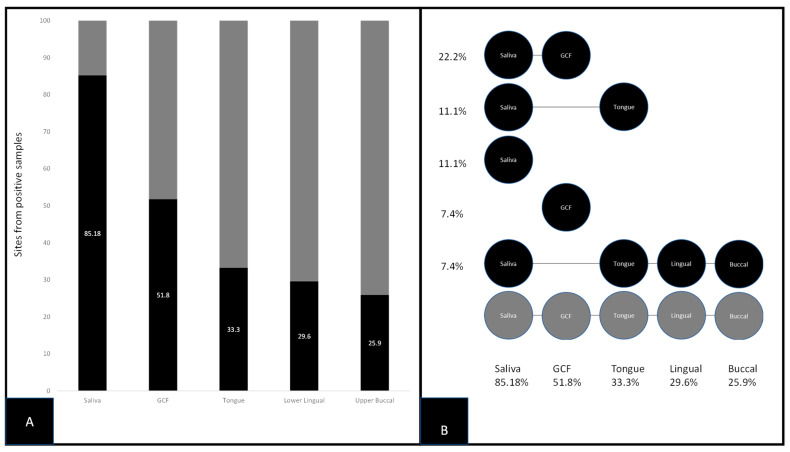
qPCR screening of samples for *S. noxia*. (**A**) Molecular screening revealed a total of *n* = 62/235 sites or 26.3% harboring SN with saliva and GCF (either alone or in combination with one or more sites) most often observed; Saliva, *n* = 23/27 or 85.18%, GCF, *n* = 14/27 or 51.%. (**B**) Analysis of site-specific data revealed most positive results were found among saliva and GCF alone or in combination, with fewer positive results observed among the tongue (33.3%), lower lingual incisor (29.6%), and upper buccal molar 25.9%.

Screening for *S. noxia* (SN) using qPCR for all sites from each patient revealed a total of *n* = 62/235 sites or 26.3% harbored this organism (Figure 3). More specifically, the site most commonly associated with an SN-positive result was saliva either alone or in combination with one or more sites, *n* = 23/27 or 85.18% (Figure 3A). The second most commonly associated site with SN-positive results was GCF either alone or in combination with saliva or other sites, *n* = 14/27 or 51.5%. Positive results for the other sites either alone or in combination with saliva or GCF were similar among the tongue (33.3%), lower lingual incisor (29.6%), and upper buccal molar (25.9%) (Figure 3B). Logistic regression of each site as a predictor of positive outcome for other sites revealed a strong odds ratio (OR = 17.8) between saliva and GCF, which was more significant than associations between any other combinations of sites (*p* = 0.00219).

To determine if any patient demographics (age, sex, race/ethnicity) or clinical information (body mass index or BMI, permanent, primary or mixed dentition, brackets or orthodontic appliances) were associated with SN positive results or site-specific positivity, negative binomial regression analysis was performed (Table 2). These data demonstrated no significant associations between SN positivity at any site and physical sample characteristics (DNA concentration or purity), patient demographics (age, BMI, sex, ethnicity) or clinical information (dentition type, presence or absence of brackets).

## 4. Discussion

The overall objective of this study was to determine which oral sites may harbor *S. noxia*, including saliva and the gingival crevice, but also additional sites, such as the dorsum of the tongue, lingual surfaces of the mandibular incisor, and buccal surface of the maxillary molars. The results of this study were successful in demonstrating that this organism may be detectable most often from samples of unstimulated saliva, comparable to findings reported from other studies [25,26]. These results also support previous reports from this group regarding the oral prevalence of this organism, based upon molecular screening of salivary samples [20,21]. In addition, these results demonstrating the presence of this organism in GCF (either alone or in combination with saliva) also support additional studies that have demonstrated the presence of this organism in significant percentages of GCF samples [4,5,6,27].

However, the results of this study also demonstrated that *S. noxia* may be present in a number of additional oral sites, such as the dorsum of the lingual surfaces of the mandibular incisor, and buccal surface of the maxillary molars, even if the GCF tested negative for this periodontal pathogen. Based upon these results, this may be the first description of the presence of this organism among additional oral sites, suggesting the presence of this organism at these specific biofilm locations may be more commonplace than previously though and may be similar to other studies of gram-negative periodontal pathogens that aggregate in developing or existing biofilms on teeth and the tongue [28,29,30]. In fact, recent studies have postulated that enrichment of periodontal pathogens may first occur in developing biofilm prior to the development of subgingival inflammation, gingivitis or periodontal disease–suggesting more detailed analysis of these oral sites may be needed in future studies [31,32,33].

These results also revealed no significant associations between clinical variables that were previously observed, such as increased prevalence in the presence of orthodontic brackets [12,13,14] and associations with higher BMI [15,16,17]. The lack of significant associations between these clinical variables and the presence of *S. noxia* may be due to the overall sample size of this pilot study. Most models for analyzing mixed predictor variables with clustered outcome data suggest a minimal sample size of at least *n* = 20 per group (sex, race/ethnicity) or category (primary, mixed, permanent dentition), which was mostly reached but not significantly exceeded in most variables and categories within this study [34,35,36]. In addition, the lack of clinical information regarding oral health and the presence of oral disease, including periodontal disease and the associated periodontal pocket depth or PPD information, may limited the inferences that can be made from this study. In future studies, an increase in overall sample size to accommodate at least *n* = 50 per group or category may be needed to more accurately evaluate any potential associations between variables and molecular screening outcomes, as well as the inclusion of additional oral health parameters, such as DMFT and PPD scores and the presence or absence of oral and systemic diseases and their corresponding medications and treatments.

The clinical and diagnostic implications of this study may provide further evidence for significance as the proximity of some of these locations (e.g., buccal surface of maxillary molars) may be associated with complications and infections of other oral sites, including the parotid gland and lymph nodes [37,38]. Although other well-defined diagnostic methods exist to sample these sites, this study provides an additional screening and diagnostic tool to complement the array of methods used to locate, identify and quantify these oral pathogens [39,40]. This type of information is useful as the extent and composition of oral biofilm sites and the relationships and interconnections with extraoral infections becomes more replete, the decisions and choices regarding treatment and interventions (including surgical modalities) may differ and outcomes may vary based upon this information in order to fully restore bone volume or tissue function [41,42,43].

In addition, as many types of systemic infections, including conditions involving immunocompromised patients (e.g., HIV or COVID-19) involve complications from oral bacteria that can colonize and translocate endotracheal intubation tubes, the ability to sample and specifically locate biofilm constituents based upon location, such as the dorsum of the tongue becomes critically important [44,45,46,47]. In fact, many other clinical settings currently provide oral sampling for other systemic conditions that include, but are not limited to, HIV and hepatitis C viruses [48,49,50,51].

Additional clinical applications may include the role and relationship of oral biofilms and site specific locations with the accuracy, measurements and subsequent success of intra-oral scanners and other methods employed in the field of digital dentistry [52,53,54]. Evidence has suggested a strong relationship between oral hygiene, biofilm accumulation and failure of many types of clinical procedures, including implants [55,56,57]. Based upon these studies, it becomes clear that a more detailed and thorough analysis of oral biofilm sites and their constituents may be critically important to ensure the success (and prevent the failure of) many other types of clinical dental treatments, as well as the outcomes of other types of respiratory and systemic infections [58,59].

However, the most significant and important clinical associations of SN remain the relationships between this organism and the presence or development of periodontal disease [60,61]. The relationship between SN and the aggressiveness of associated periodontal disease suggests that treatment protocols and clinical interventions may benefit from molecular screening to indicate the presence or levels of SN to ensure appropriate methods are used for effective treatment [62,63]. In fact, other oral and systemic conditions including oral cancer and stomatitis have been associated with periodontal disease development and progression, although the relationships with SN more specifically have yet to be evaluated [64,65]. The exploration of these relationships is of critical importance as new evidence continues to emerge that demonstrates the potential relationship between existing illness, such as oral cancer and periodontal disease, may influence both risk and progression of newly emerging pathogens, such as SARS-CoV-2 (COVID-19) [66,67].

## 5. Conclusions

Despite the limitations of the current pilot study, these results may be among the first to describe site-specific locations of *S. noxia* among various oral biofilm sites. These data may represent a significant advancement in our understanding of the sites and locations that harbor this organism, which may be important for our understanding of the prevalence and distribution of these organisms among patients of different ages undergoing different types of oral treatments, such as orthodontics.

## Figures and Tables

**Table 1:** Study sample demographics.

	Study Sample	Pediatric Clinic	Statistical Analysis
***Sex***			
***Female***	42.6% (*n* = 20/47)	49.1%	χ2 = 1.441, d.f. = 1
***Male***	57.4% (*n* = 27/47)	50.9%	*p* = 0.2300
***Race/Ethnicity***			
***White***	17.0% (*n* = 8/47)	34.6%	χ2 = 14.242, d.f. = 1
***Minority (non-White)***	83.0% (*n* = 39/47)	65.4%	*p* = 0.0002
***Hispanic***	63.8% (*n* = 30/47)	58.6%	
***Black/Asian/Other***	19.2% (*n* = 9/47)	16.8%	
***Age***			
***Average***	10.26 years	10.41 years	*p* = 0.781
***Range***	7–15 years	0–17 years	

**Table 2:** Negative binomial regression analysis of study results.

	Saliva Pr(>ǀzǀ)	GCF Pr(>ǀzǀ)	Dorsal Tongue Pr(>ǀzǀ)	Lower Lingual Pr(>ǀzǀ)	Upper Buccal Pr(>ǀzǀ)	Number of Sites Pr(>ǀzǀ)
***DNA conc.***	*p* = 0.499	*p* = 0.816	*p* = 0.862	*p* = 0.476	*p* = 0.225	*p* = 0.313
***DNA purity***	*p* = 0.619	*p* = 0.980	*p* = 0.983	*p* = 0.382	*p* = 0.591	*p* = 0.923
***Age***	*p* = 0.943	*p* = 0.639	*p* = 0.991	*p* = 0.638	*p* = 0.082	*p* = 0.181
***BMI***	*p* = 0.749	*p* = 0.967	*p* = 0.725	*p* = 0.406	*p* = 0.633	*p* = 0.595
***Sex***	*p* = 0.525	*p* = 0.414	*p* = 0.103	*p* = 0.290	*p* = 0.747	*p* = 0.317
***Ethnicity***	*p* = 0.554	*p* = 0.447	*p* = 0.082	*p* = 0.599	*p* = 0.783	*p* = 0.843
***Dentition***	*p* = 0.675	*p* = 0.826	*p* = 0.228	*p* = 0.220	*p* = 0.169	*p* = 0.331
***Brackets***	*p* = 0.975	*p* = 0.858	*p* = 0.145	*p* = 0.656	*p* = 0.939	*p* = 0.825

## Data Availability

The data presented in this study are available on request from the corresponding author. The data are not publicly available due to the study protocol data protection parameters requested by the IRB and OPRS for the initial study approval.

## References

[1] Tanner A., Bouldin H.D., Maiden M.F. (1989). Newly delineated periodontal pathogens with special reference to selenomonas species. Infection.

[2] Maiden M.F., Tanner A., Moore W.E. (1992). Identification of Selenomonas species by whole-genomic DNA probes, sodium dodecyl sulfate-polyacrylamide gel electrophoresis, biochemical tests and cellular fatty acid analysis. Oral Microbiol. Immunol..

[3] Kolenbrander P.E., Andersen R.N., Moore L.V. (1989). Coaggregation of Fusobacterium nucleatum, Selenomonas flueggei, Selenomonas infelix, Selenomonas noxia, and Selenomonas sputigena with strains from 11 genera of oral bacteria. Infect. Immun..

[4] Tanner A., Kent R., Maiden M.F., Taubman M.A. (1996). Clinical, microbiological and immunological profile of healthy, gingivitis and putative active periodontal subjects. J. Periodontal Res..

[5] Tanner A., Maiden M.F., Macuch P.J., Murray L.L., Kent R.L. (1998). Microbiota of health, gingivitis, and initial periodontitis. J. Clin. Periodontol..

[6] Socransky S.S., Haffajee A.D., Cugini M.A., Smith C., Kent R.L. (1998). Microbial complexes in subgingival plaque. J. Clin. Periodontol..

[7] Haffajee A.D., Cugini M.A., Tanner A., Pollack R.P., Smith C., Kent R.L., Socransky S.S. (1998). Subgingival microbiota in healthy, well-maintained elder and periodontitis subjects. J. Clin. Periodontol..

[8] Lundgren T., Renvert S., Papapanou P.N., Dahlén G. (1998). Subgingival microbial profile of Papillon-Lefèvre patients assessed by DNA-probes. J. Clin. Periodontol..

[9] Dibart S., Chapple I.L., Skobe Z., Shusterman S., Nedleman H.L. (1998). Microbiological findings in prepubertal periodontitis. A case report. J. Periodontol..

[10] Boström L., Bergström J., Dahlén G., Linder L.E. (2001). Smoking and subgingival microflora in periodontal disease. J. Clin. Periodontol..

[11] Craig R.G., Boylan R., Yip J., Bamgboye P., Koutsoukos J., Mijares D., Ferrer J., Imam M., Socransky S.S., Haffajee A.D. (2001). Prevalence and risk indicators for destructive periodontal diseases in 3 urban American minority populations. J. Clin. Periodontol..

[12] Anhoury P., Nathanson D., Hughes C.V., Socransky S., Feres M., Chou L.L. (2002). Microbial profile on metallic and ceramic bracket materials. Angle Orthod..

[13] Costa M.R., da Silva V.C., Miqui M.N., Colombo A.P., Cirelli J.A. (2010). Effects of ultrasonic, electric, and manual toothbrushes on subgingival plaque composition in orthodontically banded molars. Am. J. Orthod. Dentofac. Orthop..

[14] López R., Dahlén G., Retamales C., Baelum V. (2011). Clustering of subgingival microbial species in adolescents with periodontitis. Eur. J. Oral Sci..

[15] Goodson J.M., Groppo D., Halem S., Carpino E. (2009). Is obesity an oral bacterial disease?. J. Dent. Res..

[16] Cruz P., Mehretu A.M., Buttner M.P., Trice T., Howard K.M. (2015). Development of a polymerase chain reaction assay for the rapid detection of the oral pathogenic bacterium, Selenomonas noxia. BMC Oral Health.

[17] de Andrade D.R., Silva P.A., Colombo A.P.V., Silva-Boghossian C.M. (2021). Subgingival microbiota in overweight and obese young adults with no destructive periodontal disease. J. Periodontol..

[18] Rudney J.D., Chen R. (2004). Human salivary function in relation to the prevalence of Tannerella forsythensis and other periodontal pathogens in early supragingival biofilm. Arch. Oral Biol..

[19] Papaioannou W., Gizani S., Haffajee A.D., Quirynen M., Mamai-Homata E., Papagiannoulis L. (2009). The microbiota on different oral surfaces in healthy children. Oral Microbiol. Immunol..

[20] Bui Q., Nguyen C., McDaniel J., McDaniel S., Kingsley K., Howard K.M. (2017). Selenomonas noxia screening among pediatric patient samples: A pilot study. J. Oral Heal Dent. Care.

[21] McDaniel S., McDaniel J., Tam A., Kingsley K., Howard K.M. (2017). Oral Microbial Ecology of Selenemonas noxia and Scardovia wiggsiae. Microbiol. Res. J. Int..

[22] Popa C., Filioreanu A.M., Stelea C., Maftei G.A., Popescu E. (2018). Prevalence of oral lesions modulated by patient’s age: The young versus the elderly. Rom. J. Oral Rehabil..

[23] Baker J.L., Bor B., Agnello M., Shi W., He X. (2017). Ecology of the Oral Microbiome: Beyond Bacteria. Trends Microbiol..

[24] Emett J., David R., McDaniel J., McDaniel S., Kingsley K. (2020). Comparison of DNA Extracted from Pediatric Saliva, Gingival Crevicular Fluid and Site-Specific Biofilm Samples. Methods Protoc..

[25] Panda M., Rai A.K., Rahman T., Das A., Das R., Sarma A., Kataki A.C., Chattopadhyay I. (2020). Alterations of salivary microbial community associated with oropharyngeal and hypopharyngeal squamous cell carcinoma patients. Arch. Microbiol..

[26] da Silva C.M., Colombo A.V., do Souto R.M., Colombo A.P. (2005). In vivo evaluation of the effect of essential oil-containing oral strips on salivary bacteria using the checkerboard method. J. Clin. Dent..

[27] Bieri R.A., Adriaens L., Spörri S., Lang N.P., Persson G.R. (2013). Gingival fluid cytokine expression and subgingival bacterial counts during pregnancy and postpartum: A case series. Clin. Oral Investig..

[28] Chervinets V.M., Chervinets Y.V., Leont’eva A.V., Kozlova E.A., Stulov N.M., Belyaev V.S., Grigoryants E.O., Mironov A.Y. (2021). The microbiome of oral cavity patients with periodontitis, adhesive and biofilm forming properties. Klin. Lab. Diagn..

[29] Rabe A., Gesell Salazar M., Michalik S., Fuchs S., Welk A., Kocher T., Völker U. (2019). Metaproteomics analysis of microbial diversity of human saliva and tongue dorsum in young healthy individuals. J. Oral Microbiol..

[30] Ziebolz D., Söder F., Hartl J.F., Kottmann T., Rinke S., Merle C.L., Schmalz G. (2019). Prevalence of periodontal pathogenic bacteria at different oral sites of patients with tongue piercing—Results of a cross sectional study. Diagn. Microbiol. Infect. Dis..

[31] Naginyte M., Do T., Meade J., Devine D.A., Marsh P.D. (2019). Enrichment of periodontal pathogens from the biofilms of healthy adults. Sci Rep..

[32] Zaura E., Pappalardo V.Y., Buijs M.J., Volgenant C.M.C., Brandt B.W. (2021). Optimizing the quality of clinical studies on oral microbiome: A practical guide for planning, performing, and reporting. Periodontology 2000.

[33] Roldán S., Herrera D., Sanz M. (2003). Biofilms and the tongue: Therapeutical approaches for the control of halitosis. Clin. Oral Investig..

[34] Bandyopadhyay D., Galvis D.M., Lachos V.H. (2017). Augmented mixed models for clustered proportion data. Stat. Methods Med. Res..

[35] Galvis D.M., Bandyopadhyay D., Lachos V.H. (2014). Augmented mixed beta regression models for periodontal proportion data. Stat. Med..

[36] Lewis B.R., Bandyopadhyay D., DeSantis S.M., John M.T. (2017). Augmenting beta regression for periodontal proportion data via the SAS NLMIXED procedure. J. Appl. Probab. Stat..

[37] Ogle O.E. (2020). Salivary Gland Diseases. Dent. Clin. N. Am..

[38] Hammett J.T., Walker C. (2021). Sialolithiasis. StatPearls.

[39] Salgarelli A.C., Capparè P., Bellini P., Collini M. (2009). Usefulness of fine-needle aspiration in parotid diagnostics. Oral Maxillofac. Surg..

[40] Liu C.C., Jethwa A.R., Khariwala S.S., Johnson J., Shin J.J. (2016). Sensitivity, Specificity, and Posttest Probability of Parotid Fine-Needle Aspiration: A Systematic Review and Meta-analysis. Otolaryngol. Head Neck Surg..

[41] Trimarchi M., Giordano Resti A., Vinciguerra A., Danè G., Bussi M. (2020). Dacryocystorhinostomy: Evolution of endoscopic techniques after 498 cases. Eur. J. Ophthalmol..

[42] Crespi R., Capparè P., Gherlone E. (2012). Sinus floor elevation by osteotome: Hand mallet versus electric mallet. A prospective clinical study. Int. J. Oral Maxillofac. Implants.

[43] Vinci R., Teté G., Lucchetti F.R., Capparé P., Gherlone E.F. (2019). Implant survival rate in calvarial bone grafts: A retrospective clinical study with 10 year follow-up. Clin. Implant Dent. Relat. Res..

[44] Enrico G., Elisabetta P., Giulia T., Paolo C. (2021). Dentistry and Covid-19 pandemic: Operative indications post-lockdown. New Microbiol..

[45] Ejaz H., Alsrhani A., Zafar A., Javed H., Junaid K., Abdalla A.E., Abosalif K.O.A., Ahmed Z., Younas S. (2020). COVID-19 and comorbidities: Deleterious impact on infected patients. J. Infect. Public Health.

[46] Lesko C.R., Bengtson A.M. (2021). HIV and COVID-19: Intersecting Epidemics with Many Unknowns. Am. J. Epidemiol..

[47] Mohammed A.H., Blebil A., Dujaili J., Rasool-Hassan B.A. (2020). The Risk and Impact of COVID-19 Pandemic on Immunosuppressed Patients: Cancer, HIV, and Solid Organ Transplant Recipients. AIDS Rev..

[48] Parisi M.R., Tecco S., Gastaldi G., Polizzi E., D’Amicantonio T., Negri S., Gardini I., Schlusnus K., Gherlone E., Capparè P. (2017). Point-of-care testing for hepatitis C virus infection at alternative and high-risk sites: An Italian pilot study in a dental clinic. New Microbiol..

[49] Tecco S., Parisi M.R., Gastaldi G., Polizzi E., D’Amicantonio T., Zilocchi I., Gardini I., Gherlone E.F., Lazzarin A., Capparè P. (2019). Point-of-care testing for hepatitis C virus infection at an Italian dental clinic: Portrait of the pilot study population. New Microbiol..

[50] Javaid M.A., Ahmed A.S., Durand R., Tran S.D. (2016). Saliva as a diagnostic tool for oral and systemic diseases. J. Oral Biol. Craniofac. Res..

[51] Corstjens P.L., Abrams W.R., Malamud D. (2016). Saliva and viral infections. Periodontol 2000.

[52] Ferrini F., Sannino G., Chiola C., Capparé P., Gastaldi G., Gherlone E.F. (2019). Influence of Intra-Oral Scanner (I.O.S.) on The Marginal Accuracy of CAD/CAM Single Crowns. Int. J. Environ. Res. Public Health.

[53] Cattoni F., Teté G., Calloni A.M., Manazza F., Gastaldi G., Capparè P. (2019). Milled versus moulded mock-ups based on the superimposition of 3D meshes from digital oral impressions: A comparative in vitro study in the aesthetic area. BMC Oral Health.

[54] Joda T., Zarone F., Ferrari M. (2017). The complete digital workflow in fixed prosthodontics: A systematic review. BMC Oral Health.

[55] Tecco S., Grusovin M.G., Sciara S., Bova F., Pantaleo G., Capparé P. (2018). The association between three attitude-related indexes of oral hygiene and secondary implant failures: A retrospective longitudinal study. Int. J. Dent. Hyg..

[56] Bruschi G.B., Crespi R., Capparè P., Grande N., Bruschi E., Gherlone E. (2014). Radiographic evaluation of crestal bone levels of delayed implants at medium-term follow-up. Int. J. Oral Maxillofac. Implants.

[57] Polizzi E., Tetè G., Bova F., Pantaleo G., Gastaldi G., Capparè P., Gherlone E. (2020). Antibacterial properties and side effects of chlorhexidine-based mouthwashes. A prospective, randomized clinical study. J. Osseointeg..

[58] Xiang Z., Koo H., Chen Q., Zhou X., Liu Y., Simon-Soro A. (2020). Potential implications of SARS-CoV-2 oral infection in the host microbiota. J. Oral Microbiol..

[59] Kazemian H., Bourbour S., Beheshti M., Bahador A. (2017). Oral Colonization by Nosocomial Pathogens During Hospitalization in Intensive Care Unit and Prevention Strategies. Recent Pat. Antiinfect. Drug Discov..

[60] Hiranmayi K.V., Sirisha K., Ramoji Rao M.V., Sudhakar P. (2017). Novel Pathogens in Periodontal Microbiology. J. Pharm. Bioallied. Sci..

[61] Oliveira R.R., Fermiano D., Feres M., Figueiredo L.C., Teles F.R., Soares G.M., Faveri M. (2016). Levels of Candidate Periodontal Pathogens in Subgingival Biofilm. J. Dent. Res..

[62] Solomon S.M., Timpu D., Forna D.A., Stefanache M.A., Martu S., Stoleriu S. (2016). AFM comparative study of root surface morphology after three methods of scaling. Mater. Plast..

[63] Belal M.H., Watanabe H. (2014). Comparative study on morphologic changes and cell attachment of periodontitis-affected root surfaces following conditioning with CO_2_ and Er:YAG laser irradiations. Photomed. Laser Surg..

[64] Michaud D.S., Fu Z., Shi J., Chung M. (2017). Periodontal Disease, Tooth Loss, and Cancer Risk. Epidemiol. Rev..

[65] Filioreanu A.M., Popa C., Maftei G.A., Parlatescu I., Nicolae C.L., Popescu E. (2018). Migratory stomatitis–case presentation. Rom. J. Oral Rehabil..

[66] Madapusi Balaji T., Varadarajan S., Rao U.S.V., Raj A.T., Patil S., Arakeri G., Brennan P.A. (2020). Oral cancer and periodontal disease increase the risk of COVID 19? A mechanism mediated through furin and cathepsin overexpression. Med. Hypotheses.

[67] Chauhan A., Ghoshal S., Pal A. (2020). Increased susceptibility of SARS-CoV2 infection on oral cancer patients; cause and effects: An hypothesis. Med. Hypotheses.

